# Pemphigus Is not Associated with an Increased Risk of Psychiatric Disorders during the Course of the Disease

**DOI:** 10.1016/j.xjidi.2025.100432

**Published:** 2025-11-07

**Authors:** Shirin Emtenani, Salam Alfarsi, Philip Curman, Henning Olbrich, Rengin Melis Engin, Ralf J. Ludwig, Enno Schmidt

**Affiliations:** 1 Lübeck Institute of Experimental Dermatology, University of Lübeck, Lübeck, Germany; 2 Dermato-Venereology Clinic, Karolinska University Hospital, Stockholm, Sweden; 3 Department of Medical Epidemiology and Biostatistics, Karolinska Institutet, Stockholm, Sweden; 4 Dermatology and Venereology Division, Department of Medicine (Solna), Karolinska Institutet, Stockholm, Sweden; 5 Department of Dermatology, University of Lübeck, Lübeck, Germany; 6 Institute and Comprehensive Center for Inflammation Medicine, University-Hospital Schleswig-Holstein, Lübeck, Germany

**Keywords:** Pemphigus, Population-based cohort study, Psychiatric disorders, TriNetX

## Abstract

Pemphigus is a rare, severe autoimmune blistering disease caused by autoantibodies targeting desmosomal proteins, leading to intraepithelial blistering and painful erosions of the skin and mucous membranes. Although 10–15% of dermatology patients are diagnosed with psychiatric disorders, the association between pemphigus and psychiatric disorders remains unclear owing to limited large-scale evidence. We conducted a retrospective cohort study using the United States TriNetX Collaborative Network, analyzing data from over 120 million electronic health records. Adults (aged ≥18 years) with pemphigus were identified and matched 1:1 with comparators (n = 5753 individuals per group) using propensity score matching for age, sex, ethnicity, and major comorbidities. Outcomes were the incidence of psychiatric disorders diagnosed after the index event, including suicidal ideation, suicide attempts, depression, psychotic disorders, bipolar disorder, substance use disorders, anxiety, eating disorders, borderline personality disorder, attention-deficit hyperactivity disorder, and stress-related disorders, defined by International Classification of Diseases, Tenth Revision, Clinical Modification codes. Three sensitivity analyses addressed variations in follow-up time, baseline data completeness, and long-term outcome stability. No increased risk of psychiatric disorders was observed in patients with pemphigus over the course of the disease, with consistent results across all sensitivity analyses. In conclusion, these findings challenge prior assumptions and highlight the importance of large-scale, well-controlled studies in clarifying psychiatric comorbidities in autoimmune blistering diseases.

## Introduction

Pemphigus is a group of rare autoimmune blistering diseases characterized by autoantibodies that mainly targeting desmosomal adhesion molecules, specifically desmoglein 1 and 3. These autoantibodies disrupt epithelial cell–cell adhesion, causing intraepithelial blistering and erosions of the skin and/or mucous membranes (Schmidt et al, 2019). Beyond disease-related morbidity and mortality as well as the adverse effects of long-term immunosuppressive therapies (Boch et al, 2023), pemphigus significantly impairs patients' QOL, affecting their psychological and social well-being (Ghodsi et al, 2012; Mazzotti et al, 2011). Approximately 10–15% of dermatology patients have psychiatric disorders (Dalgard et al, 2018). Emerging evidence suggests elevated rates of depression, anxiety, and suicidal ideation in patients with pemphigus (Hsu et al, 2020; Kridin and Schmidt, 2021; Kridin et al, 2019, 2018; Patel et al, 2022; Tabolli et al, 2008; Wang et al, 2023; Wohl et al, 2015). However, published population-based studies have been limited by small sample sizes, single-center designs, or short follow-up periods, hindering both generalizability and long-term outcome assessment. Moreover, the role of demographic and clinical covariates in modulating psychiatric risk among patients with pemphigus is not well-understood.

To address these gaps, we conducted a retrospective cohort study using anonymized longitudinal data from the United States Collaborative Network of TriNetX, encompassing over 120 million electronic health records (EHRs) in the United States. The study aimed to evaluate the risk of psychiatric disorders, including mood disorders, psychotic disorders, and suicidal behaviors, in patients with pemphigus versus matched comparators.

## Results

### Cohort description

**Figure 1:**
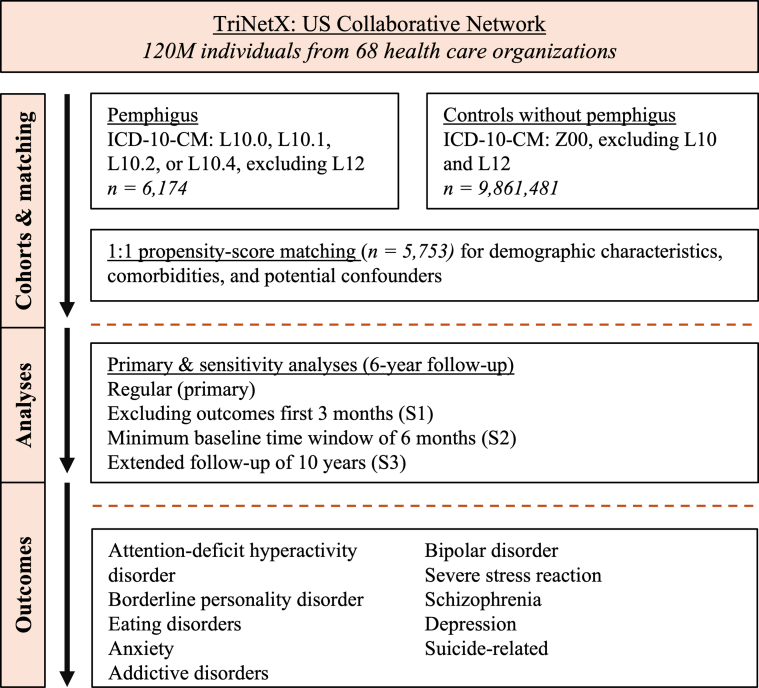
**Study flowchart**.

We retrieved 6174 EHRs from patients diagnosed with pemphigus and 9,861,481 nonpemphigus comparator EHRs from 68 healthcare organizations (Figure 1). Baseline characteristics are presented in Table 1. To enhance comparability, 1:1 propensity score matching (PSM) was performed based on age, sex, ethnicity, and major comorbidities, resulting in 5753 matched pairs (Table 1).

**Table 1:** Baseline Characteristics of Patients with Pemphigus and Comparators after Propensity Score Matching

Characteristic	Before Matching			After Matching		
	Pemphigus	Comparator	Std. diff.	Pemphigus	Comparator	Std. diff.
Number of participants	6174	9,861,481	—	5753	5753	—
Follow-up (d), median (interquartile range)	1161 (2,302)	1295 (2,393)	—	1161 (2302)	1330 (2481)	—
Age at index, y (SD)	54.6 ± 15.9	45.5 ± 18.2	0.532	54.6 ± 15.9	54.4 ± 15.9	0.008
Female (%)	55.0	51.873	0.062	55.0	54.8	0.003
Male (%)	43.2	42.9	0.007	43.2	39.4	0.078
White (%)	54.8	62.0	0.146	54.8	63.1	0.169
Diseases of the circulatory system (I00–I99, %)	20.8	22.7	0.045	20.8	20.4	0.010
Diseases of the respiratory system (J00–J99, %)	15.2	24.5	0.234	15.2	14.7	0.016
Neoplasms (C00–D49, %)	14.0	12.4	0.047	14.0	12.9	0.032
Diseases of the blood and blood-forming organs and certain disorders involving the immune mechanism (D50–D89, %)	8.4	7.7	0.026	8.8	7.7	0.024
Diseases of the musculoskeletal system and connective tissue (M00–M99, %)	22.4	30.6	0.188	22.4	21.7	0.017
Endocrine, nutritional, and metabolic diseases (E00–E89, %)	22.6	27.9	0.122	22.6	21.8	0.020
Diseases of the digestive system (K00–K95, %)	20.8	20.7	0.004	20.8	19.7	0.029

Abbreviation: Std. diff., standardized mean difference.

### Psychiatric outcomes in patients with pemphigus

**Figure 2:**
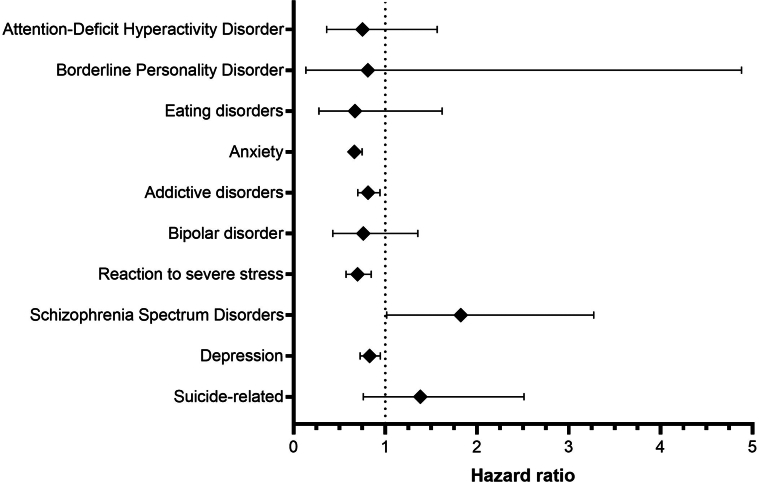
**Comparison of the risk of psychiatric disorders between patients with pemphigus and matched comparators.** Hazard ratios with 95% confidence intervals are shown. Forest plots display 95% confidence intervals; however, *P*-values were evaluated against a Bonferroni-adjusted significance threshold.

The primary analysis demonstrated a non-statistically significant elevated risk for most psychiatric outcomes in patients with pemphigus compared with matched controls. A borderline significant trend toward increased risk was observed for schizophrenia (hazard ratio [HR] = 1.82, 95% confidence interval [CI] = 1.01–3.27, *P* = .0410) and suicide-related outcomes (HR = 1.38, 95% CI = 0.76–2.51, *P* = .2858); however, these associations did not remain significant after correction for multiple comparisons (Figure 2). In contrast, the patient cohort exhibited lower risks of depression (HR = 0.83, 95% CI = 0.72–0.94, *P* = .0057), stress-related and adjustment disorders (HR = 0.69, 95% CI = 0.57–0.84, *P* = .0003), addictive disorders (HR = 0.81, 95% CI = 0.70–0.94, *P* = .0062), and anxiety (*P* < .0001). Other psychiatric outcomes, including bipolar disorder (*P* = .3528), eating disorders (*P* = .3719), borderline personality disorder (*P* = .8183), and attention-deficit hyperactivity disorder (*P* = .4459), showed no statistically significant differences between groups.

**Figure 3:**
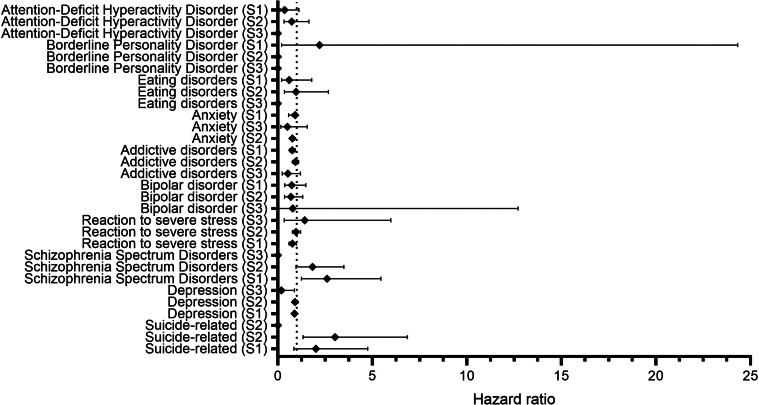
**Risks of psychiatric disorders in patients with pemphigus compared with those in matched comparators across different time windows.** The time windows are between 3 months and 6 years after diagnosis of pemphigus, within a minimal baseline time window requiring at least 1 recorded healthcare visit and over an extended 10-year observation period. Hazard ratios with 95% confidence intervals are presented.

To assess the robustness of these findings, three sensitivity analyses were conducted: S1, restricted follow-up to 3 months to 6 years after pemphigus diagnosis; S2, included only patients with at least 1 recorded healthcare encounter; and S3, extended the observation period to 10 years. All sensitivity analyses yielded results that were consistent with those of the primary findings (Figure 3).

**Figure 4:**
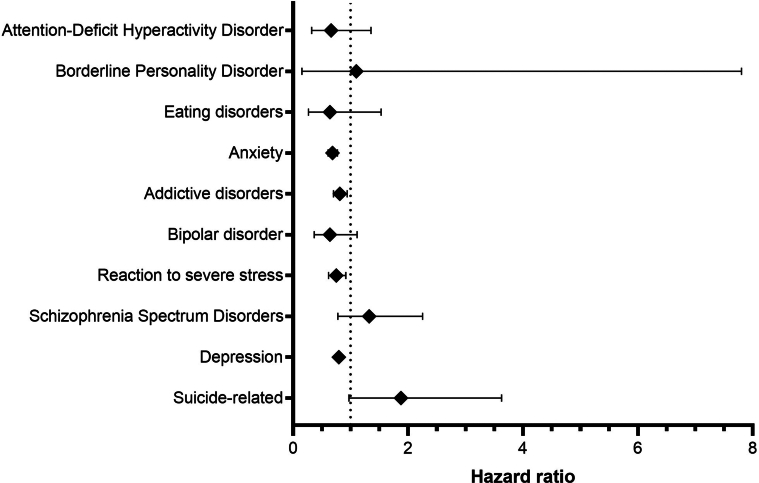
**Risks of psychiatric disorders in patients with pemphigus treated with adrenal corticosteroids compared with those in matched comparators.** Hazard ratios with 95% confidence intervals are presented.

Consistent with these findings, corticosteroid treatment in patients with pemphigus was associated with a non-significant increased risk for suicide-related outcomes (HR = 1.87, 95% CI = 0.97–3.63, *P* = .0569) and schizophrenia spectrum disorders (HR = 1.32, 95% CI = 0.77–2.25, *P* = .2973) (Figure 4).

## Discussion

This large-scale, population-based cohort study utilized the TriNetX database to investigate the potential associations between pemphigus and a range of psychiatric disorders. Contrary to prior smaller studies suggesting an elevated psychiatric risk in patients with pemphigus (Kridin et al, 2019, 2018), we found no statistically significant association between pemphigus and the incidence of several psychiatric conditions, including depression, reaction to severe stress and adjustment disorders, bipolar disorder, addictive disorders, anxiety, eating disorders, borderline personality disorder, and attention-deficit hyperactivity disorder. Notably, our analysis revealed a non-significant trend toward an increased risk of schizophrenia and suicide-related outcomes in patients with pemphigus. These findings partially align with those of Kridin et al (2019), who reported a higher prevalence of schizophrenia in patients with pemphigus (n = 1985) than in matched comparators (n = 9874), with stronger associations among females, individuals aged >60 years, and those of Jewish ethnicity. However, Kridin et al (2018) also found an increased prevalence of bipolar disorder in the same cohorts, a result not replicated in our analysis. Notably, these studies assessed the presence of psychiatric disorders and pemphigus cross-sectionally at the time of data extraction, without establishing a temporal follow-up window, limiting causal inference.

Several factors may explain these discrepancies. First, Kridin et al (2019, 2018) utilized data from an Israeli health maintenance organization, reflecting population demographics, healthcare access, and diagnostic practices different from those of the population assessed in our United States–based study. In contrast, our study leveraged the large TriNetX database, which includes EHRs from multiple healthcare systems, and implemented robust PSM for age, sex, ethnicity, and comorbidities, likely reducing residual confounding seen in earlier studies. Second, differences in data sources may have influenced the findings.

National registry data, as used by Kridin et al (2019, 2018), often provide more complete diagnostic coding and clinical detail, whereas EHR-based databases such as TriNetX offer broader population coverage but may be subject to underreporting or coding variability. Third, we excluded individuals with pre-existing psychiatric diagnoses to ensure that only incident cases were analyzed. Although this approach reduces bias from baseline differences, it may also underestimate the risk of lifetime psychiatric . In addition, our comparator group comprised individuals undergoing general examination encounters and may have included those with subclinical or undiagnosed psychiatric conditions, potentially inflating the observed rates of psychiatric outcomes and attenuating observed differences. Finally, methodological differences, including inclusion and exclusion criteria, follow-up duration, and confounder adjustment approaches, may contribute to the divergent results across studies.

Systemic corticosteroid use in patients with pemphigus was associated with a non-significant increased risk for suicide-related conditions and schizophrenia, suggesting that treatment regimens may contribute to psychiatric outcomes.

Our findings should be interpreted in light of several limitations. Reliance on International Classification of Diseases, Tenth Revision, Clinical Modification codes may introduce misclassification bias, and some psychiatric diagnoses, particularly those managed outside participating healthcare settings, may be underreported. Unmeasured confounders, such as socioeconomic status, disease severity, or access to mental health services, could influence the observed associations. As part of the PSM process, a subset of patients with pemphigus was excluded to achieve optimal covariate balance between exposed and unexposed groups. This exclusion is a known limitation of matching-based study designs and may reduce the generalizability of our findings to the broader pemphigus population. However, it strengthens internal validity by improving group comparability and reducing confounding. Finally, differences in healthcare-seeking behavior between groups may affect detection of psychiatric conditions. Future research should incorporate patient-reported outcomes, longitudinal psychological assessments, and data from diverse global populations to validate and extend these findings. The possible association between pemphigus and psychiatric disorders cannot be fully ruled out, because these conditions may be underdiagnosed by dermatologists if not evaluated using appropriate assessment tools during patient visits.

In conclusion, our study suggests that pemphigus is not significantly associated with an increased risk of most psychiatric disorders compared with matched comparators, challenging earlier reports based on smaller or differently designed samples. These results underscore the value of large-scale, rigorously matched cohort studies in clarifying disease–psychiatric comorbidity associations. Although routine psychiatric screening in patients with pemphigus remains important, our study suggests that it may not be necessary beyond the standard care applied to individuals with chronic illnesses.

## Materials and Methods

### Study design and database

We conducted a global, population-based retrospective cohort study using PSM, following previously described methods (Emtenani et al, 2024; Ludwig et al, 2025). Data for this retrospective study were retrieved from approximately 120 million EHRs in the United States Collaborative Network of TriNetX, accessed in July 2024 (Figure 1). The index event was defined as the first recorded diagnosis used for cohort inclusion.

### Study population and eligibility criteria

The study included 5345 patients with pemphigus aged ≥18 years, identified using the following International Classification of Diseases, Tenth Revision, Clinical Modification codes: pemphigus vulgaris (L10.0), pemphigus foliaceous (L10.2), pemphigus erythematosus (L10.4), and pemphigus vegetans (L10.1). Patients with diagnoses of pemphigoid (L12) were excluded. The comparator cohort comprised individuals with an International Classification of Diseases, Tenth Revision, Clinical Modification code for “Encounter for general examination without complaint, suspected or reported diagnosis” (Z00), excluding those with any diagnosis of pemphigus or pemphigoid (L10 or L12).

### Covariates and outcomes

Covariates included age at index, sex, ethnicity, and comorbidities: circulatory (I00–I99), respiratory (J00–J99), neoplastic (C00–D49), blood (D50–D89), musculoskeletal (M00–M99), endocrine (E00–E89), and digestive (K00–K95) diseases. TriNetX provides standardized race and ethnicity categories harmonized across participating institutions. The reported categories included White, Black or African American, Asian, Hispanic or Latino, and other/unknown. Race and ethnicity were collected to describe the demographic composition of the study population and to evaluate potential disparities in disease prevalence and psychiatric comorbidities among patients with pemphigus.

The primary outcome was the incidence of psychiatric disorders occurring after the index event, including suicidal ideations (R45.851); suicide attempt (T14.91); recurrent major depressive disorder (F33); depressive episode (F32); schizophrenia, schizotypal, delusional, and other nonmood psychotic disorders (F20–F29); bipolar disorder (F31); mental and behavioral disorders due to psychoactive substance use (F10–F19); other anxiety disorders (F41); eating disorders (F50); borderline personality disorder (F60.3); attention-deficit hyperactivity disorder, predominantly inattentive type (F90.0); and reaction to severe stress and adjustment disorders (F43).

The primary analysis evaluated outcomes from 1 day after the first occurrence of the index event through a 6-year follow-up period. Three sensitivity analyses were conducted: S1, follow-up restricted to 3 months to 6 years after the index event; S2, requiring at least one healthcare visit within the 6 months before the index event; and S3, a 10-year observation period. Patients with preindex psychiatric diagnoses were excluded. In addition, adrenal corticosteroid treatment (VS:HS050) was also evaluated as a covariate.

### Statistical analysis

Differences between cohorts were assessed using the log-rank test. HRs with 95% CIs were calculated using univariate Cox proportional hazards regression. The proportional hazards assumption was tested using scaled Schoenfeld residuals. Kaplan–Meier survival analysis was used to evaluate time-to-event data, with pairwise log-rank comparisons for each psychiatric outcome. All analyses were conducted after PSM. Two-tailed *P* < .05 were considered statistically significant. A Bonferroni correction was applied to account for multiple comparisons (α_adjusted_ = 0.007).

## Ethics Statement

This study involved the secondary analysis of deidentified data and did not include direct interaction or intervention with human subjects. All data were deidentified in accordance with section §164.514(a) of the Health Insurance Portability and Accountability Act Privacy Rule. The deidentification process was validated by a qualified expert, as outlined in section §164.514(b)(1), with the most recent determination issued in December 2020. As such, institutional review board approval was not required.

## Data Availability Statement

The data that support the findings of this study are available from the corresponding author upon request.

## ORCIDs

Shirin Emtenani: http://orcid.org/0000-0002-7965-8045

Philip Curman: http://orcid.org/0000-0003-2051-8491

Henning Olbrich: http://orcid.org/0000-0003-0863-7148

Ralf J. Ludwig: http://orcid.org/0000-0002-1394-1737

Enno Schmidt: http://orcid.org/0000-0002-1206-8913

## Conflict of Interest

RJL has received financial support for travel from TriNetX. The remaining authors state no conflict of interest.
